# CRISPR screening identifies T cell-intrinsic regulators of CD3-bispecific antibody responses

**DOI:** 10.3389/fimmu.2022.909979

**Published:** 2022-08-05

**Authors:** Ryan D. Molony, Theresa Funk, Gina Trabucco, Erik Corcoran, David Ruddy, Malini Varadarajan, GiNell Elliot, Michelle Piquet, Joni Lam, Matthew J. Meyer, Hui Qin Wang, Sema Kurtulus, Haihui Lu

**Affiliations:** Novartis Institutes for BioMedical Research, Cambridge, MA, United States

**Keywords:** Bispecific antibodies, T cells, immunotherapy, CRISPR screening, mTOR, EP300

## Abstract

CD3-engaging bispecific antibodies (BsAbs) enable the formation of an immune synapse between T cells and tumor cells, resulting in robust target cell killing not dependent on a preexisting tumor specific T cell receptor. While recent studies have shed light on tumor cell-specific factors that modulate BsAb sensitivity, the T cell-intrinsic determinants of BsAb efficacy and response durability are poorly understood. To better clarify the genes that shape BsAb-induced T cell responses, we conducted targeted analyses and a large-scale unbiased *in vitro* CRISPR/Cas9-based screen to identify negative regulators of BsAb-induced T cell proliferation. These analyses revealed that CD8+ T cells are dependent on CD4+ T cell-derived signaling factors in order to achieve sustained killing *in vitro*. Moreover, the mammalian target of rapamycin (mTOR) pathway and several other candidate genes were identified as intrinsic regulators of BsAb-induced T cell proliferation and/or activation, highlighting promising approaches to enhancing the utility of these potent therapeutics.

## Introduction

The advent of CD3-engaging bispecific antibody (BsAb) therapeutics and mechanistically related chimeric antigen receptor (CAR) T cell therapies has revolutionized the field of immunoncology (1, 2). The potential of BsAb-based approaches to T cell activation and tumor cell killing have been explored for decades (3, 4), and the initial approvals arrived with the 2010 European Union approval of catumaxomab for malignant ascites and the 2014 US Food and Drug Administration (FDA) approval of blinatumomab for Ph-negative relapsed and refractory acute lymphoblastic leukemia (ALL) patients (5, 6). In addition, there are numerous BsAb molecules in ongoing clinical trials, with multiple BCMAxCD3 and CD20xCD3 BsAb demonstrating impressive responses and nearing approval (7). These BsAb constructs function by forming a transient direct immune synapse between T cells and cells expressing the target antigen of choice (8), bypassing normal interactions between the T cell receptor (TCR) and antigen-presenting major histocompatibility complex (MHC) molecules to induce robust polyclonal cytotoxic T cell activation and consequent cytolytic tumor clearance. The establishment of novel approaches to generating a diverse array of BsAbs has the potential to further extend the clinical utility of these molecules (9), underscoring the need for further efforts to understand and optimize their activity.

While BsAb treatment has achieved promising efficacy in patients, therapeutic resistance and treatment-related adverse events remain as important clinical concerns (10–12). Moreover, the durability of BsAb treatment-associated responses in certain therapeutic contexts is uncertain (13), and the role of cytokine release in the context of *in vivo* BsAb-induced T cell cytotoxicity has been reported to be dispensable (14). Further study of the correlates of durable T cell cytotoxicity and the relative contributions of the CD4+ and CD8+ T cell compartments following BsAb treatment are thus urgently needed, as they may have the potential to greatly expand the clinical utility of this therapeutic modality. Genome-wide CRISPR screens have recently been performed in target tumor cell lines to clarify the genetic basis for the emergence of therapeutic resistance to BsAb treatment (15). However, no such screens have been reported in BsAb-activated T cells to date. The development of CRISPR/Cas9-based “Guide Swap” strategies (16) and similar techniques have enabled genome-wide CRISPR-based screening in primary T cells (17), highlighting an opportunity to apply a similar strategy to more fully probe T cell-specific determinants of BsAb efficacy.

Herein, we conducted a large-scale CRISPR/Cas9 screen in primary human T cells with the goal of identifying the genes that most potently regulate T cell responses to repeated BsAb-induced activation. Moreover, we explored the relative contributions of CD4+ and CD8+ T cells to the potency and durability of BsAb-induced cytotoxicity using CD3-engaging BsAb constructs targeting a series of different tumor antigens. Through these assays, we were able to clarify multiple cell-intrinsic and cell-extrinsic mechanisms that ultimately govern the ability of these T cells to engage and kill target tumor cells, suggesting promising targets for future clinical study in this immunotherapeutic context.

## Materials and methods

### Cell culture

KMS-11 (multiple myeloma) cells were cultured in RPMI-1640 (Thermo Fisher Scientific, USA) supplemented with 20% FBS, and were maintained at a density of 0.5x10^6^-2.0x10^6^ cells/mL. Primary T cells were cultured in RPMI-1640 supplemented with 10% FBS, L-glutamine, Non-essential amino acids, HEPES, sodium pyruvate, and β-mercaptoethanol, and were maintained at a density of 0.25x10^6^-1.0x10^6^ cells/mL unless otherwise noted. All cells were cultured in humidified incubators at 37°C with 5% CO_2_.

### T cell isolation

Peripheral blood mononuclear cells (PBMCs) were isolated from peripheral blood of healthy human donors *via* Ficoll density gradient centrifugation. T cells were then isolated from these PBMCs by negative selection using the Pan T Cell Isolation Kit, human (Miltenyi Biotec, USA) following the manufacturer’s protocol. For appropriate experiments, CD4+ and CD8+ T cells were positively enriched from these isolated T cells using CD4 and CD8 microbeads (Miltenyi Biotec) according to the manufacturer’s protocols.

### Cell staining and flow cytometry

For cell-surface staining, at the indicated time points, cells were collected, washed with PBS, and stained with a LIVE/DEAD Fixable Violet Dead Stain Kit (Thermo Fisher Scientific) according to the manufacturer’s instructions. Cells were then washed with cold autoMACS^®^ Running buffer (FACS buffer; Miltenyi Biotec) prior to resuspension in antibody cocktails containing appropriate fluorescently conjugated antibodies specific for human cell-surface antigens. Antibodies used in this analysis included anti-CD4-PE-Cy7, anti-CD8-APC-Cy7, anti-CD45RO-FITC, anti-CD27-BV650, anti-CD62L-BV768, anti-CD3-PerCP-Cy5.5, anti-CD69-APC (1:100; all from Biolegend, USA). Cells were stained for 20 minutes on ice, after which they were washed twice with FACS buffer prior to resuspension in a 200 uL volume for acquisition.

To stain for intracellular phosphorylated proteins, viability and cell surface staining were completed as above. Cells were then fixed using cold Cytofix buffer (BD Biosciences) for 30 minutes on ice, after which they were resuspended in Perm/Wash Buffer III (BD Biosciences) for 30 min on ice, washed twice with cold stain buffer (2% FBS in PBS), and resuspended in stain buffer containing anti-pan-S6-AF647, anti-pS6-AF647, or an AF647-conjugated isotype control antibody (Cell Signaling Technology, USA). Cells were stained for 30 minutes at room temperature, after which they were washed twice with FACS buffer prior to resuspension in a 200 uL volume for acquisition.

All flow cytometry data were acquired on an LSR Fortessa flow cytometer (BD Biosciences, USA), with a minimum of 10,000 gated events being acquired per sample. Captured data were analyzed using the FlowJo software package.

### RTCC and repeat challenge assays

T cells were plated in 96-well plates (5x10^4^ cells/well) or 24-well plates (2x10^5^ cells/well) together with appropriate target tumor cells at a 1:2 T cell: Target cell ratio, and indicated doses of antigen-specific or control BsAbs. Cells were then incubated for 1-4 days, after which cells were processed for flow cytometry, target cell killing assay, or repeat challenge setup. Target cell killing was analyzed using a Bio-Glo Luciferase Assay System (Promega, USA) based upon provided protocols. Briefly, 100 uL of the prepared Bio-Glo reagent was added per well of 96-well plates, and plates were mixed for 2 minutes. Luciferase activity was then measured using an EnVision plate reader (PerkinElmer, USA).

For repeat challenge assays, a subset of T cells in 24-well plates were analyzed by flow cytometry as above, and the number of live T cells and tumor cells per sample was determined. Remaining cells were then washed with FACS buffer, and were replated as above (2x10^5^ cells/well). Additional target tumor cells were added to a final concentration of 4x10^5^ cells/well, and fresh BsAb was added at an experimentally appropriate concentration. Cells were then incubated as above for up to 4 days. This process was repeated for the duration of repeat challenge assays. In appropriate experiments, cells were treated with the indicated concentrations of inhibitor compounds at the indicated doses for the duration of the repeat challenge assay.

### LegendPlex cytokine analysis

Levels of T cell-derived cytokines in cell supernatant samples were measured with a bead-based LegendPlex Kit (Human T Helper Cytokine Panel Version 2, BioLegend) according to the manufacturer’s instructions. An LSR Fortessa flow cytometer (BD Biosciences) was used for data acquisition, while data analysis was performed using the LegendPlex data analysis suite (BioLegend).

### CRISPR/Cas9-mediated gene editing

Primary T cells (0.5x10^6^ cells/mL) were isolated as above and combined with Dynabeads Human T-Activator CD3/CD28 beads (Thermo Fisher Scientific) at a 2:1 bead to T cell ratio for 3 days in antibiotic-free T cell media. Cas9 ribonucleoprotein (RNP) complexes were then prepared by combining 1 µL of tracrRNA (100 µM in nuclease-free duplex buffer; IDT, USA) with 1 µL of the appropriate crRNA 100 µM in nuclease-free duplex buffer; IDT) for each sample. Tubes were heated to 95°C for 2 min, cooled to room temperature for 5 min, and combined with a Cas9 master mix containing 0.79 µL of Cas9 (61 µM; IDT) and 2.21 µL of buffer T from the Neon Transfection System 100 µL kit. RNPs were incubated at room temperature for 20 min, after which 0.6x10^6^ T cells per sample were resuspended in the RNP-containing solution and immediately transfected using the Neon Electroporation System using the following settings: 1600 V, 10 ms, 3 pulses. Cells were then transferred to 24-well plates and incubated for 3 days, after which Dynabeads were removed, and cells were counted prior to use in downstream experiments.

The crRNAs used in this study are listed in **Supplementary Table 1**.

### Pooled CRISPR screening

The CRISPR/Cas9-mediated gene editing for pooled CRISPR screening was achieved *via* the previously published ‘Guide Swap’ approach. T cells were isolated from two healthy donors (500,000 cells/donor) and activated using Dynabeads (2:1 beads to T cells) as above in 225 cm^2^ tissue culture flasks. After 1 day, cells were counted, resuspended in antibiotic-free T cell media in 6-well plates (2x10^6^ cell/well), and transduced with pooled lentiviral particles (150 µL/well) (15) by spinning plates for 90 min at 1200 *xg.* Half of the virus-containing supernatant was then aspirated and replaced with 2 mL of antibiotic-free T cell media. Following a 2-day incubation, cells were electroporated with Cas9 RNP complexes containing a Nontargetting sgRNA as above. Cells were then replated in 75 cm^2^ tissue culture flasks (0.25x10^6^/mL) and incubated for 3 days. Remaining Dynabeads were then removed to complete T cell library preparation.

For screening, T cell libraries were either used directly as a mixed CD4/CD8 pool under mixed culture conditions, or were separated to isolate individual CD4 and CD8 T cell libraries for each donor. Supplemental IL-2 (10 ng/mL) was added to all individual CD4 and CD8 samples, but was omitted from mixed T cell samples. T cells in these three pools were then challenged with KMS11-Luc target cells (1:2 E:T ratio) and the anti-BCMA BsAb (3 nM) as above, with challenges being repeated every 3-4 days for up to 31 days. At each rechallenge time point, samples were analyzed for cell surface staining as above, and were assessed for red fluorescent protein (RFP) positivity to confirm the relative frequency of lentivirally-transduced cells. At each time point (Days 0, 4, 7,11, 14, 18, 21, 25, 28, 31), cell pellets containing at least 10^7^ cells were collected for each sample and snap-frozen. At all times during the screening process, T cells were cultured in quantities sufficient to ensure a minimum of 200x coverage for each of the 65,000 guides included in the library.

DNA sequencing, bioinformatics analyses, and enriched hit identification were conducted as reported previously (15). Ordered lists of top hits in CD4+ and CD8+ T cells were determined by averaging all fold-change (FC) values for samples collected on Day 14+ (CD4 T cells) or Day 18+ (CD8 T cells) of the screen relative to baseline (Day 0) values. The top 100 hits in each T cell type were used for Hallmark Pathway Analyses. Twenty four genes were selected for validation from among the top 50 hits in CD4+ and CD8+ T cells based on putative pathway annotations and corresponding literature review.

## Results

### Evaluation of longitudinal T cell responses to BsAb-mediated activation

**Figure 1:**
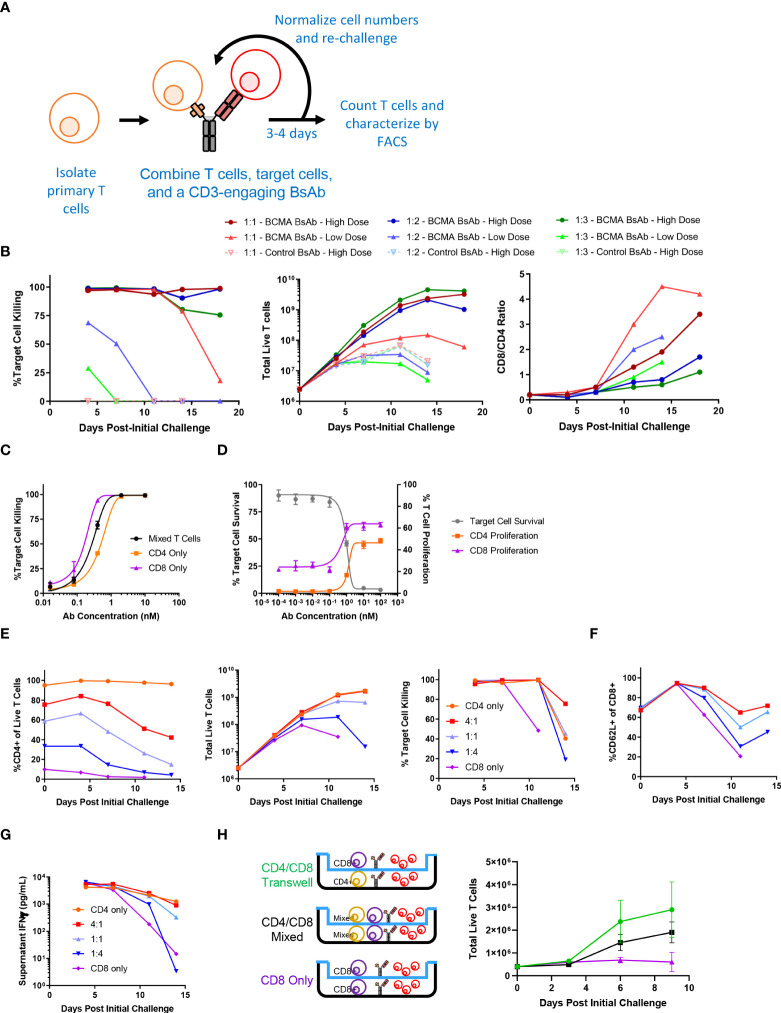
Sustained CD4+ T cell help is necessary for long-term CD3-engaging bispecific antibody-mediated CD8+ T cell proliferation and tumor cell killing *in vitro*. **(A)** Schematic overview of the long-term repeat challenge assay system. **(B)** A long-term repeat challenge assay was established using KMS11 target cells and a BCMAxCD3 or control CD3-engaging BsAb. Challenge was repeated every 3-4 days until killing and/or proliferation activity were absent. The efficiency of BsAb-mediated KMS11 target cell killing by T cells was quantified *via* flow cytometry to assess the dependence of these activities on BsAb dose and effector to target cell ratio (E:T). The ratio of CD8/CD4 cells was also evaluated over time (High BsAb dose = 3 nM, Low BsAb dose = 0.5 nM). **(C)** CD4+ and CD8+ T cells were negatively enriched from healthy human donor PBMCs. CD4+, CD8+, or a 1:1 mixture of CD4+ and CD8+ T cells were combined with KMS11 tumor cells containing a luciferase reporter construct (E:T – 1:1) and the indicated dose of a BCMAxCD3 BsAb for 3 days, followed by the analysis of target cell killing. **(D)** CFSE-labeled T cells and KMS11 target cells were combined for 3 days, after which CD4 and CD8 T cell proliferation and target cell killing were analyzed by flow cytometry. **(E)** CD4+ and CD8+ T cells were negatively enriched from healthy human donor PBMCs, and were then either maintained separately or combined at defined ratios (4:1, 1:1, 1:4) in a long-term repeat challenge assay with a BCMAxCD3 BsAb and KMS11 target cells. Cell killing was monitored over time. **(F)** CD62L+ CD8+ T cell frequencies were monitored over time. **(G)** Supernatant IFNγ levels were measured by ELISA in samples collected at each re-challenge time point. **(H)** CD4+ and CD8+ T cells were negatively enriched from two healthy donors and used in a Transwell repeat challenge assay as shown, with proliferation of cells in the upper chamber being quantified over time. Data in **(B)** are representative plots from a single donor. All experiments were repeated a minimum of three times, except for **(H)** which was repeated two times.

To explore the longitudinal responses of primary human T cells following CD3-engaging BsAb-mediated activation, we developed an *in vitro* repeat challenge assay system (**Figure 1A**). We chose to model the BsAb-mediated response using a CD3-engaging BCMA (B cell maturation antigen)-specific BsAb as multiple clinical molecules in this class are being developed and have shown promising anti-myeloma efficacy in clinical trials. A tool BsAb PL33 was made with an anti-BCMA Fab (clone J6M0 (18)) and an anti-CD3 scFv (clone SP34 (19)) on a human IgG backbone. KMS11, a multiple myeloma cell line that expresses BCMA was used as the target cell line, and it was engineered to overexpress luciferase which was then used to measure cell survival. Briefly, primary T cells derived from healthy donor peripheral blood mononuclear cells (PBMCs) were combined with luciferized target cell KMS11 (KMS11-luc) multiple myeloma (MM) cells and the PL33 BsAb at a range of effector to target cell (E:T) ratios and antibody concentrations. Twice per week, fresh target cells and fresh BsAb were added at defined ratios until T cells were no longer able to efficiently kill target cells. Under these conditions, we observed marked T cell proliferation and target cell killing that was sustained for up to 19 days (**Figure 1B**). Notably, killing activity was more rapidly lost when E:T ratio values and BsAb doses were lower, suggesting that both of these variables shape T cell responses in this assay system, making this well-suited to the exploration of determinants of durability of T cell responses to BsAb-mediated activation. Interestingly, when the relative frequencies of CD4+ and CD8+ T cells were evaluated at each repeat challenge time point, we found that CD8+ T cells were increasingly more abundant with time such that CD8/CD4 ratio values rose from < 0.2 at baseline to between 2 and 5 by day 19 in a BsAb dose- and E:T ratio-dependent manner (**Figure 1B**). This suggests that BsAb activates CD8+ T cell proliferation much more robustly than CD4+ T cells.

### CD8+ T cells require help from CD4+ T cells to mediate prolonged BsAb-mediated tumor cell killing *in vitro*

To directly assess the differences in BsAb-mediated activation dynamics of CD4+ and CD8+ T cells, we separately evaluated their response characteristics. Both CD4+ and CD8+ cells were able to efficiently kill KMS11-luc tumor cells in a BsAb dose-dependent manner, although we observed a slight shift in the half-maximal effective concentration (EC50) values for these two cell types (**Figure 1C**), suggesting that CD8+ T cells may more efficiently kill tumor cells in response to suboptimal BsAb doses, in line with their prototypical effector roles. Importantly, we also found that while both of these cell types were able to readily proliferate following BsAb-mediated activation, CD8+ T cells exhibited a higher degree of proliferation at all tested BsAb doses relative to CD4+ T cells (**Figure 1D**), potentially explaining their observed tendency to outcompete CD4+ T cells over the course of a repeat challenge experiment (**Figure 1B**). Interestingly, despite their ability to kill target tumor cells and to proliferate more readily than CD4+ T cells in a single round killing assay, in a repeat challenge assay system we found that CD8+ T cells exhibited markedly reduced proliferation and target cell killing over time in the absence of CD4+ cells, with the overall maintenance of CD8+ T cell responses being dependent upon the starting ratio of CD4:CD8 T cells (**Figure 1E**). Consistent with the observed loss of cytotoxic activity, we found that CD8+ cells that lacked access to CD4+ T cell-derived help more rapidly lost expression of the central memory marker proteins CD62L and CD27, and that production of IFNγ was maximal when CD4+ T cells were also present in the cultures (**Figures 1F, G** and **Supplementary Figure 1A**). Similar results were also observed using the same BCMA-targeting BsAb and LP-1 target tumor cells, a HER-2-targeting BsAb (with the Her2 antigen binding fragment, Fab, from Trastuzumab) and HER-2-expressing HCC1954 tumor cells, or a CD19-targeting BsAb and CD19-expressing Ocily19 cells, suggesting that our results are not restricted to this specific cell line model or target antigen (**Supplementary Figure s 2B-G**).

To determine whether the mechanisms whereby CD4+ T cells were able to support CD8+ T cell responses in a repeat challenge assay were contact dependent, we conducted a Transwell experiment in which CD4 and CD8 T cells from a given healthy donor were used in a repeat challenge assay in which direct contact between these two cell types was not possible (**Figure 1H**). CD8+ T cells were able to proliferate equally well in this assay setting regardless of whether or not CD4+ T cells were separated by the permeable Transwell membrane interface, suggesting that CD4+ derived secreted factors are sufficient to sustain prolonged CD8+ T cell responsiveness to BsAb-mediated activation. Cell culture supernatants were then collected at the end of the first challenge in a repeat challenge assay and analyzed with a multiplexed bead-based kit, revealing that CD4+ T cells secreted much higher levels of immunomodulatory cytokines including both interleukin (IL)-2 and IL-10 relative to CD8+ T cells (**Supplementary Figure 2A**). While IL-2 is well-known to regulate T cell proliferation (20), IL-10 is typically regarded as an anti-inflammatory cytokine although it has been reported to exhibit suppressive and beneficial effects in a dose- and context-dependent manner (21, 22). Knocking out both the *IL10RA* and *IL2RG* genes *via* a CRISPR/Cas9 approach in primary CD8+ T cells markedly impaired their proliferation relative to that of CD4+ T cells when compared to CD8+ T cells treated with a non-targeting sgRNA (**Supplementary Figure 2B**), suggesting that CD4-derived IL-2 and IL-10 may play an important role in sustaining BsAb-mediated CD8+ T cell proliferation and tumor cell killing. In further support of this possibility, when CD8+ T cells were cultured in a repeat challenge assay in the presence of a fixed dose of exogenous recombinant cytokines, only the addition of both IL-2 and IL-10 in combination significantly enhanced the proliferation of these cells, whereas other cytokines that were differentially abundant when comparing CD4+ and CD8+ T cells such as IL-17 and IL-22 had no beneficial effect on CD8+ T cell proliferation (**Supplementary Figure 2C**). However, further work will be needed to systematically define the cytokines necessary for CD4+ T cell-mediated support of CD8+ T cell responses in the context of BsAb activation, as many factors beyond those included here have the potential to shape these responses.

### Knockout of negative regulators of TCR-dependent T cell activation prolongs BsAb-induced CD8+ T cell proliferation and killing

**Figure 2:**
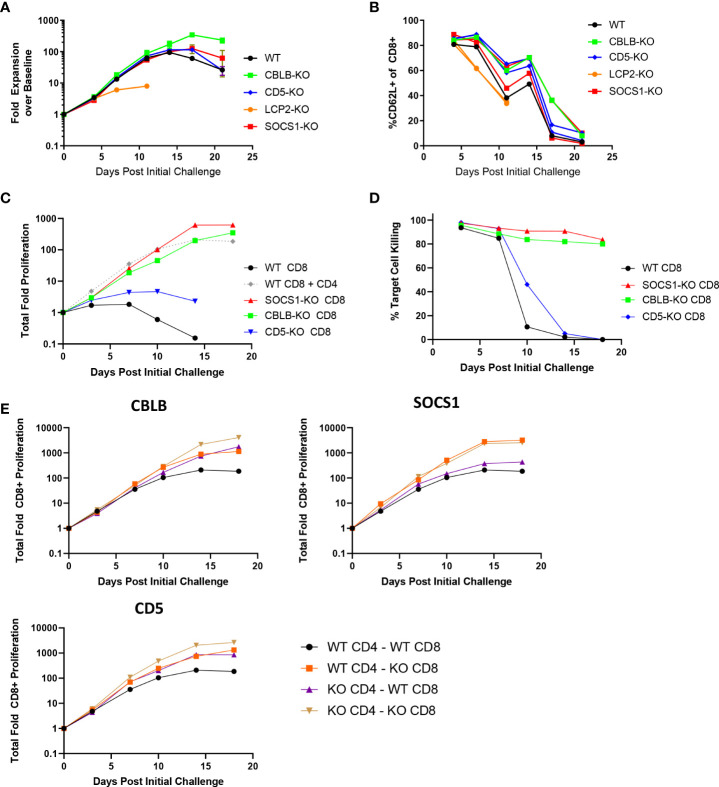
CBLB, CD5, and SOCS1 serve as negative regulators of CD4 and CD8 T cell responses to BsAb-mediated activation. **A-B.** A CRISPR/Cas9 approach was used to knock out the indicated genes in healthy donor T cells, after which a long term repeat challenge assay was performed as in **Figure 1** for the indicated T cell populations. Cellular proliferation and CD62L-positivity were measured over time *via* flow cytometry. **C-D.** A CRISPR/Cas9 approach was used to knock out the indicated genes in purified CD8+ T cells, which were then used in a long term repeat challenge assay as above to monitor proliferation and target cell killing over time. All populations other than the gray dashed line in (C) correspond to CD8+ cells without access to CD4+ T cell help. **E**. The indicated genes were knocked out in both CD4+ and CD8+ T cells, which were then combined together to test cell-intrinsic and cell-extrinsic benefits of the knockout of these three genes to the proliferation of CD8+ T cells, revealing that SOCS1 exclusively provided cell-intrinsic benefits, whereas CBLB and CD5 knockout provided cell-intrinsic and -extrinsic benefits. Experiments were repeated in duplicate using T cells from two donors.

To explore the effects of the knockout of individual genes on BsAb-induced T cell responses as a proof of concept for our screen and to examine the impact of the CD8+ T cell-intrinsic and -extrinsic determinants of prolonged BsAb-mediated proliferation and killing activity *in vitro*, we next conducted the targeted CRISPR/Cas9-mediated knockout of known negative regulators of TCR-induced T cell activation identified in a previous CRISPR screen (17), including *CBLB, SOCS1*, and *CD5.* When these three genes were knocked out in a mixed population of CD4+ and CD8+ T cells in a repeat challenge assay, moderate improvements in overall T cell proliferation and maintenance of naïve/central memory phenotypes (CD62L+) were observed (**Figures 2A, B**). Knockout of *CBLB* produced the most robust phenotypes in this assay system, whereas the deletion of the known positive regulator of TCR-induced T cell activation *LCP2* markedly blunted T cell responses.

Notably, the knockout of *CBLB* and *SOCS1* in CD8+ T cells grown in the absence of CD4+ help obviated their requirement for such help (**Figures 2C, D**), enabling these CD8+ T cells to undergo sustained proliferation and target cell killing for the duration of an 18-day repeat challenge assay. We then explored the roles of these regulatory genes as CD8-intrinsic regulators of responses to BsAb-mediated activation by knocking them out in either CD4+ or CD8+ T cells from a given healthy donor and combining these cell subsets together for use in a repeat challenge assay in which overall CD8+ T cell proliferation was the primary readout. CD8+ T cell proliferation was enhanced when *CBLB* and *CD5* were knocked out in both CD4+ and CD8+ cells, suggesting that these genes serve as both CD8-intrinsic and CD8-extrinsic regulators of the BsAb-induced proliferation of these cells (**Figure 2E**). The loss of *CD5* expression in this setting had a more pronounced impact on CD8+ T cell proliferation than it did on total T cell population (**Figure 2A**), possibly owing to the fact that CD4+ T cells were predominant in the mixed T cell population, potentially masking the effects of *CD5* knockout on the smaller CD8+ T cell population in this setting. In general, the benefits of knocking out *CD5* were less pronounced than those observed following the loss of *CBLB* or *SOCS1*. Notably, the knockout of *SOCS1* in CD4+ cells failed to benefit CD8+ cell proliferation, suggesting that SOCS1 functions exclusively as a CD8-instrinsic regulator of CD8+ cell activation in this assay context, potentially through the modulation of cellular responses to immunomodulatory cytokines including IL-2 and potentially other cytokines such as IL-10, given its function as a suppressor of cytokine-induced JAK/STAT signaling (23). Additional experiments will be necessary to define the specific CD8-intrinsic signaling requirements that are reduced in the absence of SOCS1-mediated negative feedback in order to guide the generation of more robust BsAb-induced CD8+ effector T cells responses.

### Pooled CRISPR screening identified key regulators of T cell responses to BsAb-mediated activation

**Figure 3:**
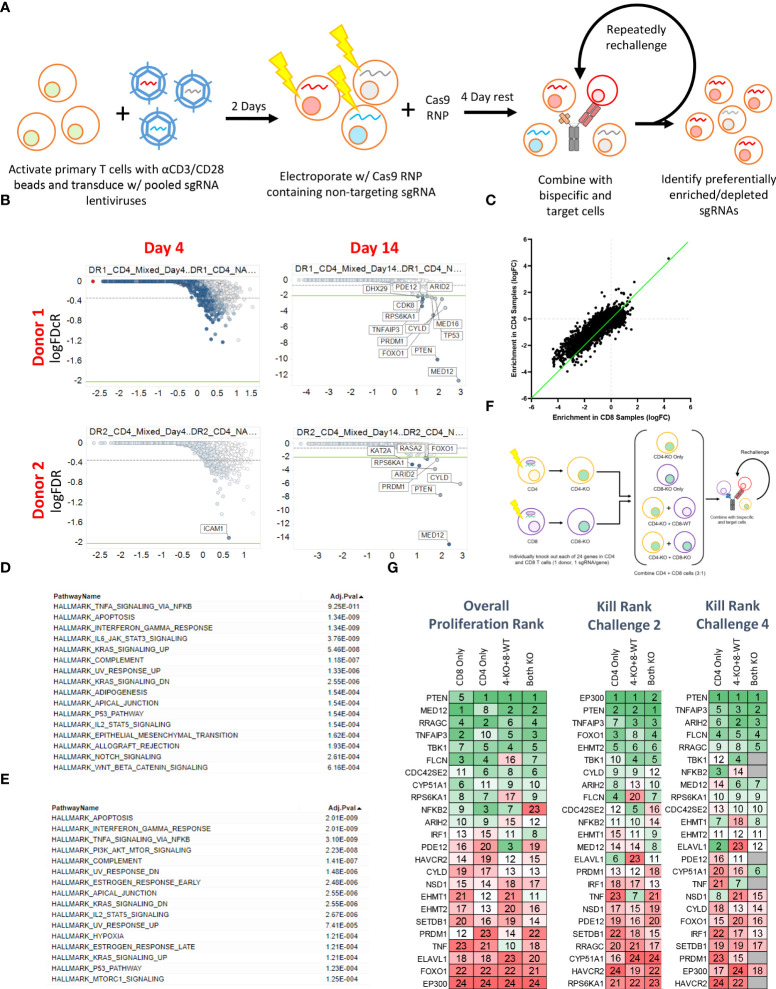
Pooled CRISPR screening enables the identification of genes regulating CD4 and CD8 T cell responses to BsAb-mediated activation. **(A)** Overview of the assay process. Briefly, T cells from two healthy donors were transduced with a lentiviral library encoding sgRNAs for approximately 1/3^rd^ of the human genome (10 sgRNAs/gene). Two days later, cells were electroporated with Cas9 complexed with a non-coding sgRNA. Cells were then repeatedly challenged with KMS11 target tumor cells every 3-4 days, with DNA being collected from T cells at each repeat challenge timepoint to assess sgRNA enrichment or depletion. **(B)** Representative plots of hit identification on days 4 and 14 of the challenge process in CD4+ T cells from each of the two screened donors. **(C)** Relative enrichment of different hits in CD4+ and CD8+ T cell samples, with each point corresponding to a single gene. The green line represents the y = x axis. **D-E.** Hallmark pathway analyses were used to assess pathway enrichment for the top 100 screen hits identified in **(D)** CD8+ T cells and **(E)** CD4+ T cells. **(F)** Screen validation strategy overview. **(G)** High-level validation result overview showing that the loss of different genes had differential impacts on long-term proliferation (after four challenges), killing after two challenges, and killing after four challenges. Genes were ranked from 1-24, with 1 indicating more killing or proliferation (as appropriate). Screening data were derived from two donors with two sets of CD4 and CD8 T cell samples per donor. Screening validation in **(G)** was performed in duplicate using T cells from a single donor.

To more broadly explore the T cell-intrinsic factors that regulate proliferation and killing activity in response to repeated BsAb-mediated activation, we next performed a pooled CRISPR screen. For this experiment, we transduced cells with a previously published lentiviral library encoding sgRNAs specific for one-third of the human genome (15, 24), after which cells were electroporated with Cas9 ribonucleoproteins (RNPs) containing a non-targeting sgRNA that was exchanged for lentivirally-encoded sgRNAs within cells through a “Guide Swap” approach (16) (**Figure 3A**). The resultant T cell was then used in a screen in which CD4+, CD8+, or mixed T cell populations from two donors were repeatedly challenged with KMS11-luc target cells and the BCMA-targeting BsAb PL33 (3 nM), with DNA being collected from cells twice per week to monitor the selective enrichment or deletion of cells transduced with particular sgRNAs (**Supplementary Figure 3A**). The frequency of red fluorescent protein (RFP)+ T cells was also measured over time as a marker for lentiviral transduction to gauge the overall relative proliferation of lentivirally transduced cells (**Supplementary Figure 3B**).

Sequencing of DNA samples from T cells collected at early and late time points revealed negligible specific sgRNA enrichment on Day 4 of this screen, whereas by Day 14 several hits were clearly evident based on the relative enrichment of T cells in which the indicated genes had been knocked out (**Figure 3B**; **Supplementary Tables 2-4**). When we ranked the top 50 hits observed in CD4+ and CD8+ T cells at late time points (Day 14+) based on logFC values, we found that both *CBLB* and *CD5* were among the top 20 hits in CD8+ cells but were absent from the list of top hits in CD4+ cells (**Supplementary Figure 3C**), consistent with the more profound impact of these genes in CD8+ cells observed above (**Figure 2**), and further validating the results of our screen. While distinct hits were detected in CD4+ and CD8+ T cells in this screen, we observed significant overlap with respect to the hits detected in these two cell types, and we found that when the logFC enrichment values for these hits were compared between these two cell subsets, they were largely similar in magnitude, with some hits exhibiting apparent CD4+ T cell bias as evidenced by their deviation from this trend (**Figure 3C**). This suggests that the majority of the screened genes play a similar regulatory role in the context of the BsAb-mediated activation of CD4+ and CD8+ T cells. Even so, when we conducted a Hallmark pathway enrichment analysis of the top hits from these two cell types, we found that while most enriched pathways were shared for both CD4+ and CD8+ cells, only CD4-associated screen hits were enriched for the ‘PI3K AKT mTOR signaling’ and ‘mTORC1 signaling’ pathways (**Figures 3D, E**), highlighting a potentially distinct role for the mTOR signaling machinery in BsAb-stimulated CD4+ T cells.

To validate and expand upon the results of our screen, we next selected 24 hits for validation from among the top 50 hits based on their magnitude of enrichment and annotated associations with pathways of interest (including the mTOR pathway) and individually knocked them out in CD4+ and CD8+ cells from a single healthy donor and used these cells either individually or mixed together in a repeat challenge assay (**Figure 3F**). These 24 hits were then ranked according to the maximal observed proliferation of the different T cell populations in which these genes had been knocked out and according to observed target cell killing at both early (Day 7) and late (Day 14) time points (**Figure 3G**). Almost all of these 24 genes were associated with significant increases in CD4+ and CD8+ T cell proliferation relative to WT cells (**Supplementary Figures 4A, B**), although a subset of hits (*FLCN, RRAGC, ARIH2*) was associated with a reduction in relative CD8+ proliferation relative to that of CD4+ cells (**Supplementary Figure 4C**), suggesting that these genes may play more critical role in CD4+ T cell proliferation, activation as compared to that of CD8+ cells.

### Loss of EP300 potentiates tumor cell killing by T cells

**Figure 4:**
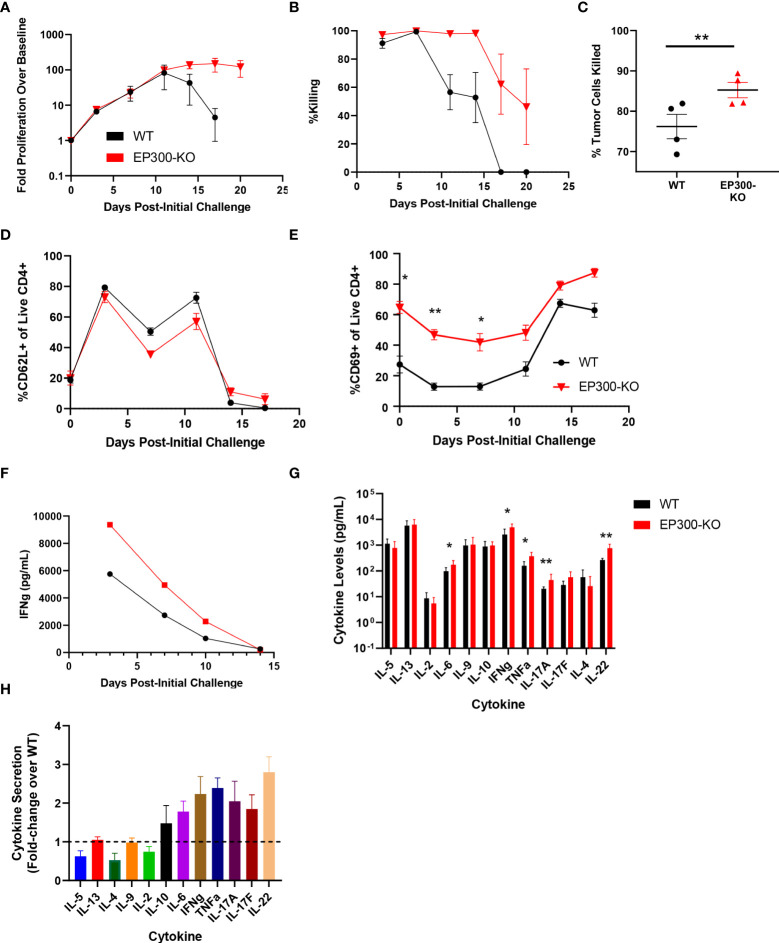
*EP300* encodes a negative regulator of BsAb-mediated T cell activation and cytotoxicity. EP300-KO CD4+ T cells and WT CD4+ T cells were used in a long-term repeat challenge assay. **(A)** T cell proliferation over time was monitored by flow cytometry. **(B)** KMS11 target cell killing by the indicated T cell populations was monitored over time by flow cytometry. **(C)** Target cell killing was measured based on luciferase activity during Challenge 2 after 24 h, revealing more complete target cell killing by *EP300-*KO CD4+ T cells at this time point. **(D)** The frequency of CD62L+ CD4+ T cells in the indicated groups was monitored over time by flow cytometry. **(E)** The frequency of CD69+ CD4+ T cells in the indicated groups was monitored over time by flow cytometry. **(F)** Supernatant IFNγ levels were measured over time by ELISA in the indicated treatment cells. **(G)** A LegendPlex cytokine assay was used to quantify the levels of the indicated cytokines in supernatants collected from WT or EP300-KO CD4+ T cells at the end of Challenge 1. **(H)** Cytokine levels in *EP300-*KO CD4+ T cell samples in **(G)** were normalized to levels in WT cell samples. All validation data in **(A-E)** and **(G-H)** were generated using T cells from four separate donors, and are representative of two experiments. Data in **(F)** are derived from a single donor and are representative of two experiments. *P<0.05, **P<0.01; Data were analyzed using two-way ANOVAs or Paired Student’s t-test with multiple testing correction as appropriate. Data are presented as the mean with standard error of the mean.

Interestingly, while *EP300* was identified as a hit in our overall screen, in our validation assay this hit showed limited benefit on long term T cell expansion and was associated with poor BsAb-mediated target cell killing at late time points but with markedly enhanced killing activity at early time points (**Figure 3G**). *EP300* encodes a histone acetyltransferase (HAT) that has previously been shown to suppress effector T cell antitumor responses by modulating regulatory T cell activity (25). To assess whether *EP300* plays a similar role in the context of BsAb-mediated T cell activation, we knocked out this gene in CD4+ T cells from four healthy donors and found that while EP300-KO was not associated with any improvement in maximal T cell proliferation relative to WT cells, it was associated with the prolonged T cell population maintenance and killing activity (**Figures 4A, B**). Consistent with the results of the 24-gene validation experiment, *EP300-*KO CD4+ T cells killed target tumor cells significantly more quickly than did WT cells (**Figure 4C**). While loss of *EP300* did not substantially alter CD62L expression over time on CD4+ T cells (**Figure 4D**), it did markedly increase the expression of the early activation marker CD69 on these cells at all time points (**Figure 4E**). These EP300-KO T cells additionally secreted higher levels of IFNγ (**Figure 4F**) and other effector cytokines associated with the Th1 and Th17 lineages including TNFα, IL-17A, and IL-17F (**Figures 4G, H**). These results suggested that a loss of *EP300* expression may skew CD4+ T cells towards a more effector-like Th1 and/or Th17 phenotype that is conducive to more robust BsAb-mediated target tumor cell killing, but that fails to benefit long-term T cell proliferation.

### Disruption of mTOR activation enhances the sustained proliferation, killing, and memory-like phenotypic characteristics of CD4+ T cells following BsAb-mediated activation

**Figure 5:**
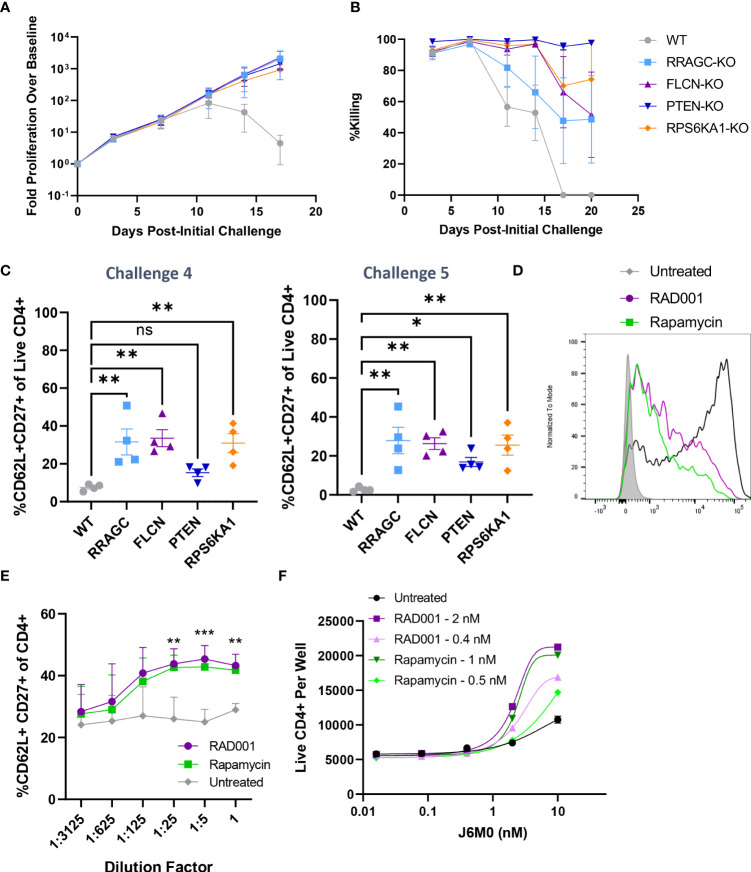
Knockout of mTOR-related genes favors memory-like cell expansion in response to BsAb-mediated challenge. **(A)** *FLCN, RRAGC, PTEN*, or *RPS6KA1* were knocked out in healthy CD4+ T cells from four healthy human donors and used in a repeat challenge assay system. The proliferation of the indicated T cell populations was monitored over time by flow cytometry. **(B)** KMS11 target cell killing by the indicated T cell populations was monitored over time by flow cytometry. **(C)** The frequency of young-like CD62L+ CD27+ CD4+ T cells in the indicated treatment groups was assessed at the end of Challenge 4 and Challenge 5. **(D)** Levels of p-S6 following treatment with RAD001/Rapamycin at the tested dose range (0.8 nM RAD001, 0.4 nM Rapamycin) in CD4+ T cells following BsAb-mediated activation were measured as a readout for mTORC1 inhibition. **(E)** A single-round BsAb challenge assay was performed using CD4+ T cells treated with the indicated dose of RAD001 or Rapamycin, with the frequency of young-like CD62L+ CD27+ CD4+ T cells in the indicated treatment groups being assessed by flow cytometry. Statistics are shown for RAD001 vs. Untreated. **(F)** Cells were treated with the indicated RAD001 and Rapamycin doses, with a single round BsAb challenge assay being performed using the indicated doses of the J6M0 tool BsAb and with CD4+ T cell counts being measured by flow cytometry. All validation data in **(A-C)** were generated using T cells from four separate donors, and are representative of two experiments. Data in **(D-F)** are derived from two donors and are representative of at least two experiments. **P<0.01, ***P<0.001; Student’s t-test with FDR correction or one-way ANOVAs with Holm-Sidak multiple comparisons testing, as appropriate. Data are presented as the mean with standard error of the mean. NS, non-significant.

Furthermore, our validation results suggested that the loss of four mTOR-related genes (*PTEN, FLCN, RRAGC*, and *RPS6KA1*) was associated with enhanced long-term proliferation and target tumor cell killing (**Figure 3G**). While *PTEN* is a well-characterized tumor suppressor gene (26), the implications of its loss and the loss of these other mTORC1-associated genes in the context of prolonged BsAb-mediated T cell activation remain to be defined. Given that we found these hits to be CD4-skewed in our overall CRISPR screen (**Supplementary Figure 3C**), we next knocked them out in healthy donor CD4+ T cells and found that the loss of any of these four genes was sufficient to enhance overall T cell proliferation and target cell killing in our repeat challenge assay system (**Figures 5A, B**; **Supplementary Figure 4D**). Importantly, the loss of these genes enhanced the cell surface expression of CD62L and CD27 on T cells at later time points, consistent with a more central memory-like phenotype having enabled these cells to undergo sustained proliferation (**Figure 5C**). Chemical inhibition of the mTOR pathway activity with low doses of rapamycin and RAD001 has been shown to preferentially inhibit mTORC1 at lower doses (27), and which we confirmed was able to downmodulate mTORC1 activation as evidenced by reduced phospho-S6 levels in our co-culture assay system (p-S6, **Figure 5D**). This treatment was sufficient to phenocopy the effects of the loss of any of these four genes, significantly enhancing CD4+ T cell CD62L- and CD27-positvity (**Figure 5E**) and proliferation (**Figure 5F**) in a manner dependent upon both inhibitor and BsAb dose in a single round target cell killing assay. Knockout of *FLCN* and *RRAGC* also prolonged target cell killing mediated by a Her2-specific BsAb to some extent, albeit with more pronounced donor-to-donor variability (**Supplementary Figure 4E**).

When a qPCR array was used to evaluate the relative expression of mTOR signaling pathway-related genes in WT and *FLCN-*KO CD4+ T cells, WT but not *FLCN-*KO cells were found to downregulate insulin signaling-related genes (*INS, INSR, IGFBP3, IGF1*) at later time points when their proliferative activity had been largely lost (**Supplementary Figure 5A**). Given the importance of insulin signaling as a driver of T cell glycolytic activity and proliferation (28, 29) together with the known role of mTORC1 signaling as an inhibitor of insulin signaling (30), these results suggest that the disruption of BsAb-induced mTOR activation may interfere with a negative feedback pathway that constrains insulin-regulated T cell expansion, thus benefitting prolonged T cell activation and target tumor cell killing.

IL-10 has recently been identified as an inhibitor of mTOR signaling in myeloid cells (31). Given that we identified IL-10 as a CD4-derived cytokine linked to more robust CD8+ T cell proliferation in the presence of CD4+ cells (**Supplementary Figures 2B, C**), we lastly tested the ability of exogenous IL-10 to suppress mTORC1 activation following BsAb-mediated T cell activation. IL-10 increased the frequency of CD62L+CD27+ cells through IL-10R-specific signaling pathways (**Supplementary Figures 5B, C**). As mTORC1 inhibition was sufficient to facilitate sustained BsAb-induced T cell proliferation in our repeat challenge assay system, this suggests that CD4-derived IL-10 may suppress mTORC1 activation in both CD4+ and CD8+ T cells, better enabling them to undergo sustained proliferation following BsAb-mediated activation.

## Discussion

The development of CD3-engaging bispecific antibodies capable of forming a transient immune synapse between T cells and target cells holds great promise as a therapeutic approach to tumor clearance (1, 2), yet relatively little is known at present regarding T cell-dependent factors that govern the magnitude and durability of these BsAb-induced antitumor responses. We found that both CD4+ and CD8+ T cells were capable of mediating tumor cell killing following BsAb-mediated activation. Strikingly, while CD8+ T cells exhibited more robust short-term cytolytic activity, the duration of such activity and associated proliferation were markedly hampered in the absence of CD4+ T cell-derived cytokines, including IL-2 and IL-10. Through a CRISPR screening assay, we identified *EP300* as a gene that, when knocked out in T cells, enhanced effector-like activity and killing at the expense of longevity. Conversely, we identified several mTOR-related genes (*FLCN, RRAGC, PTEN, RPS6KA1*) that were negative regulators of durable BsAb-induced CD4+ T cell proliferation. The knockout of these genes or the inhibition of mTOR signaling activity were sufficient to enhance memory-like CD4+ T cell responses and to provide prolonged help to CD8+ T cells to achieve target tumor cell killing, underscoring a central role for mTOR signaling in this context. Overall, these results highlight several avenues for future preclinical investigation that may hold promise to guide the design of more efficacious BsAb treatment regimens or to identify patients most likely to benefit from such treatment.

While often regarded as primarily helper cells responsible for cytokine production and the coordination of complex immunological responses, CD4+ T cells also possess endogenous cytolytic potential (32, 33). Consistently, we found that both CD4+ and CD8+ T cells were able to facilitate short-term BsAb-induced target tumor cell lysis, with CD4+ T cell-mediated target cell lysis only being slightly less robust than that for CD8+ T cells. Strikingly, however, while CD4+ T cells were able to mediate sustained tumor cell killing in a BsAb repeat challenge assay in the absence of CD8+ T cells, that was not the case for CD8+ T cells, with CD8+ cells rapidly losing their proliferative and cytolytic capacity in the absence of CD4 cell-derived soluble factors. While further work will be needed to fully clarify the cytokine(s) most critical for CD8+ responses in this context, we found that signaling through both the IL-10 receptor and the IL-2 receptor common γ chain were associated with such proliferation. IL-2 is well-established as a potent mediator of T cell survival and proliferation, and in line with the immodulatory roles of both IL-2 and IL-10, we found that the exogenous addition of these two cytokines, but not other CD4+ T cell-derived cytokines such as IL-17 and IL-22, was sufficient to enhance the proliferation of CD8+ T cells in the absence of direct CD4+ help. Consistently, the knockout of SOCS1 in CD8+ T cells, a potent inhibitor of IL-2 family cytokine signaling (23), obviated the need for CD4+ help to sustain long-term BsAb-mediated target tumor cell killing by CD8+ T cells, whereas its knockout in CD4+ cells failed to impact CD8+ responses in this assay context. In contrast, the knockout of CD5 and CBLB, which negatively regulate TCR signaling (17, 34, 35), enhanced CD8+ T cell proliferation in a long-term repeat challenge assay in both a cell-intrinsic and cell-extrinsic manner, suggesting that general enhancement of BsAb-induced TCR activation in either T cell subset can augment the overall magnitude of the resultant T cell responses. These findings align well with the results of our screen, which identified largely similar hits in CD4+ and CD8+ T cells, with slight preferential enrichment for hits including *CD5* and *CBLB* in the CD8+ compartment.

Many of the most robust hits identified in CD4+ T cells through our *in vitro* BsAb CRISPR screen were associated with the activity of the mTOR pathway, which functions as a central regulator of the balance between catabolic and anabolic activity within cells. *EP300*, which encodes the p300 histone acetyltransferase, has previously been shown to inhibit effector T cell responses (25). Consistent with such a mechanism, we found the knockout of *EP300* to augment the effector-like properties of BsAb-activated CD4+ T cells, resulting in more efficient activation and target cell killing at early time points following BsAb addition together with more robust effector cytokine production at the expense of impaired long-term cellular proliferation. Our finding that *EP300-*KO CD4+ T cells exhibited more robust effector- rather than memory-like activity, while consistent with the prior study discussed above and with evidence that p300 can favor immunosuppression (25, 36), were surprising given the results of our screen, underscoring the need for further study of the relationship between BsAb-induced activation, autophagy, and nutrient availability. Future analyses of p300-induced chromatin modifications and the relationship between immunosuppression and mTOR activity may provide key insights into viable approaches to enhancing the efficacy or durability of BsAb-induced responses.

Four of the most robust hits identified through our screening approach were *PTEN, FLCN, RRAGC*, and *RPS6KA1*, with the knockout of each of these genes favoring better long-term T cell proliferation and target cell killing in the context of a long-term bispecific killing assay. Given its thoroughly studied tumor suppressor functions (26), the identification of *PTEN* as a negative regulatory gene in this therapeutic context is perhaps unsurprising. Folliculin, encoded by *FLCN*, functions as an activator of RagC, encoded by *RRAGC*, thereby promoting mTORC1 activation in response to changing intracellular amino acid availability (37). Consequently, the loss of either *FLCN* or *RRAGC* would be expected to yield similar phenotypic outcomes associated with impaired mTORC1 activation, as observed in our validation assays. *RPS6KA1* encodes a member of the family of ribosomal protein S6 kinases, which are activated downstream of mTOR and coordinate certain mTOR-dependent intracellular responses (38). Given that *RPS6KA1-*KO T cells were phenotypically similar to *FLCN-*KO and *RRAGC-*KO T cells in the context of long-term BsAb-mediated target cell killing, this may suggest a model wherein *FLCN/RRAGC* function as key mediators of mTORC1 activation within T cells following BsAb-induced activation, while *RPS6KA1* coordinates downstream mTORC1-induced signaling responses that ultimately limit the durability of central memory-like immune cell phenotypes. While the specific downstream mechanisms associated with this pathway remain to be fully clarified, our qPCR array data suggest that the prolonged expression of genes associated with insulin signaling may contribute to this phenotype, as the sustained expression of these genes was evident in *FLCN-*KO but not WT CD4+ T cells in a long-term repeat challenge assay. This is consistent with the reported role of insulin signaling in sustaining the proliferation and glycolytic activity of T cells *in vitro* and *in vivo (* 28, 29), and its inhibition by mTORC1 (30).

These findings align well with seminal reports demonstrating that mTOR inhibition can favor memory-like CD8+ T cell differentiation at the expense of effector T cell responsivity (39). While such immunosuppression may be deleterious in many immunological contexts, the partial suppression of mTOR signaling activity in the context of BsAb-mediated polyclonal T cell activation may be beneficial by partially restraining the resultant T cell response to strike an appropriate balance between effector activity and memory differentiation. Indeed, the maintenance of mTOR activity within an optimal range has previously been proposed to be integral to striking an appropriate balance between short- and long-term T cell responses (40, 41). Our findings suggest that suboptimal mTOR inhibition can tweak this balance such that T cells exhibit a sustained central memory-like phenotype compatible with both BsAb-mediated cytotoxicity and prolonged proliferation. In contrast, the complete ablation of mTOR activity in these cells *via* the knockout of the RAPTOR adaptor protein resulted in a rapid loss of both proliferation and cytotoxicity (data not shown), underscoring the importance of incomplete mTOR inhibition. While IL-10 is generally regarded as an immunosuppressive cytokine, in the context of robust polyclonal BsAb-mediated T cell activation, we found IL-10 signaling to be associated with better prolonged T cell proliferation, central memory-like T cell phenotypes, and reduced mTOR activity. Given this evidence and recent reports highlighting the ability of IL-10 signaling to suppress mTOR activity in myeloid cells (31), we speculate that IL-10 may serve as an endogenous moderator of BsAb-induced T cell responses, constraining them within an appropriate range conducive to prolonged therapeutic efficacy. In addition, there is prior evidence that CD4+ T cell-derived IL-10 can support CD8+ T cell memory responses (42, 43). While prolonged IL-10 release has been reported in patients undergoing blinatumomab treatment (44), further work will be necessary to establish whether this cytokine is ultimately beneficial or deleterious to BsAb-mediated therapeutic efficacy in a more complex *in vivo* setting.

Overall, our results highlight several promising areas for future research aimed at improving and appropriately tuning the efficacy and durability of anti-tumor immune responses following the administration of CD3-engaging BsAbs. Additional research examining how factors including age, disease status, transcriptional regulation, and the CD4/CD8 balance influence BsAb efficacy have the potential to aid in the further refinement of this therapeutic modality. Importantly, further exploration is warranted to better define the context-specific role of mTOR signaling in this therapeutic setting and to clarify the link between such signaling and the cytolytic activity of CD4+ and CD8+ T cells *in vivo.*

## Data availability statement

The original contributions presented in the study are included in the article/**Supplementary Material**, further inquiries can be directed to the corresponding author.

## Author contributions

RM, TF, GT, and EC performed experiments and corresponding data analysis. RM, TF, GT, MM, HW, SK, and HL designed experiments. DR, MV, MP, and GE conducted DNA sequencing, hit identification, and supporting bioinformatics analyses. JL produced BsAb constructs for experimental use. All authors contributed to the article and approved the submitted version.

## Funding

This study received funding from NIBR. The funder was not involved in the study design, collection, analysis, interpretation of data or the writing of this article. The governing body at NIBR supported the decision to submit for publication. All authors declare no other competing interests.

## Acknowledgments

The authors wish to thank Melissa Ramones, Rosalie deBeaumont, Brian Granda, Adwait Oka, Tony D’Alessio, Eric Billy, Stan Ye, Si-Qi Liu, Sunyoung Jang, Uli Bialucha, Kevin Marks, Jeffrey Engelman, and Glenn Dranoff for experimental input, project oversight, reagents necessary for CRISPR screening, and technical support in the execution of this study.

## Conflict of interest

Authors RM, TF, GT, EC, DR, MP, JL, HW, and HL are currently employed by the Novartis Institutes for Biomedical Research (NIBR). The following authors were previously employed by NIBR when they were involved with the work described herein: MV (currently employed by Arbor Biotechnologies), GE (currently employed by Foghorn Therapeutics), MM (currently employed by Bristol Myers Squibb), and SK (currently employed by 2seventy Bio).

## Publisher’s note

All claims expressed in this article are solely those of the authors and do not necessarily represent those of their affiliated organizations, or those of the publisher, the editors and the reviewers. Any product that may be evaluated in this article, or claim that may be made by its manufacturer, is not guaranteed or endorsed by the publisher.
