# Supplementary material for: CRISPR screening identifies T cell-intrinsic regulators of CD3-bispecific antibody responses
Source: Front Immunol. 2022 Aug 5;13:909979. doi: 10.3389/fimmu.2022.909979 (PMC9388929; doi:10.3389/fimmu.2022.909979)
Supplement: Supplementary file 1 [file DataSheet_1.pdf]

SUPPLEMENTARY FIGURES

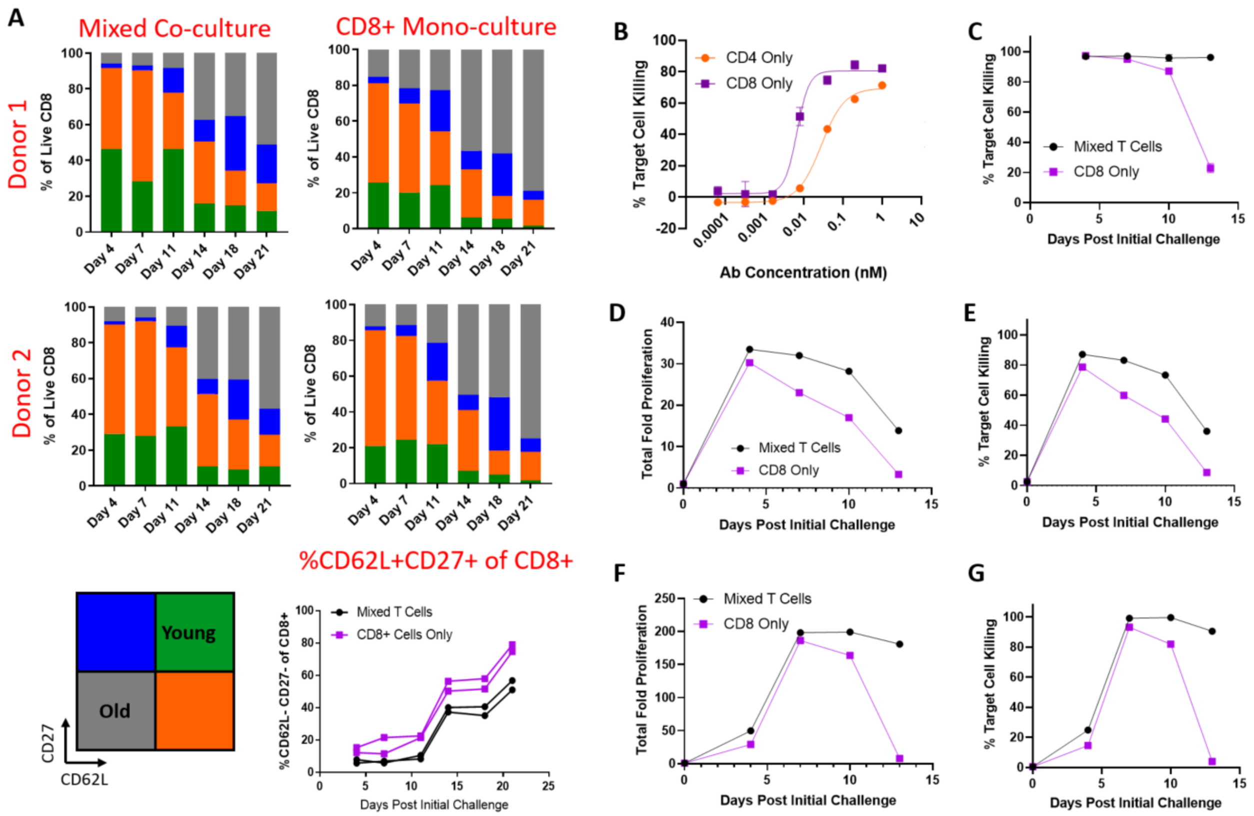

Supplementary Figure 1. Differences in CD4+ and CD8+ T cell response dynamics are observed for multiple BsAb and target cell combinations. A. CD8+ T cells from two healthy human donors were cultured either alone or together with CD4+ T cells at a 4:1 CD4:CD8 ratio in a repeat challenge assay with KMS11-luc target cells and the J6M0 target antibody. Cells were cultured for 21 days, with fresh target cells and BsAb being added every 3-4 days. Cell surface CD27 and CD62L expression was measured by flow cytometry at the indicated time points. **B.** CD8+ or CD4+ T cells were combined with HCC1954 tumor cells containing a luciferase reporter construct (E:T – 1:1) and the indicated dose of a Her2-specific CD3-engaging BsAb. After 3 days, luciferase activity in individual wells was used to quantify tumor cell killing. **C.** A long-term re-challenge assay was conducted using LP-1 cells as targets and CD8+ T cells cultured on their own or mixed with CD4 cells at a 4:1 CD4:CD8 ratio. Killing was then monitored over time by flow cytometry. **D-E.** T cells were co-cultured with HCC1954 tumor cells and a Her2-specific BsAb as in (B) in a repeat challenge assay, after which T cell proliferation (D) and target cell killing (E) were monitored over time by flow cytometry. **F-G.** T cells were co-cultured with Ocily19 tumor cells and CD19-specific BsAb in a repeat challenge assay, with T cell proliferation (F) and target cell killing (G) being monitored over time. All experiments were repeated a minimum of two times. Data in C-G depict results from individual T cell donors, but are representative of a minimum of three independent experimental replicates performed with T cells from multiple donors.

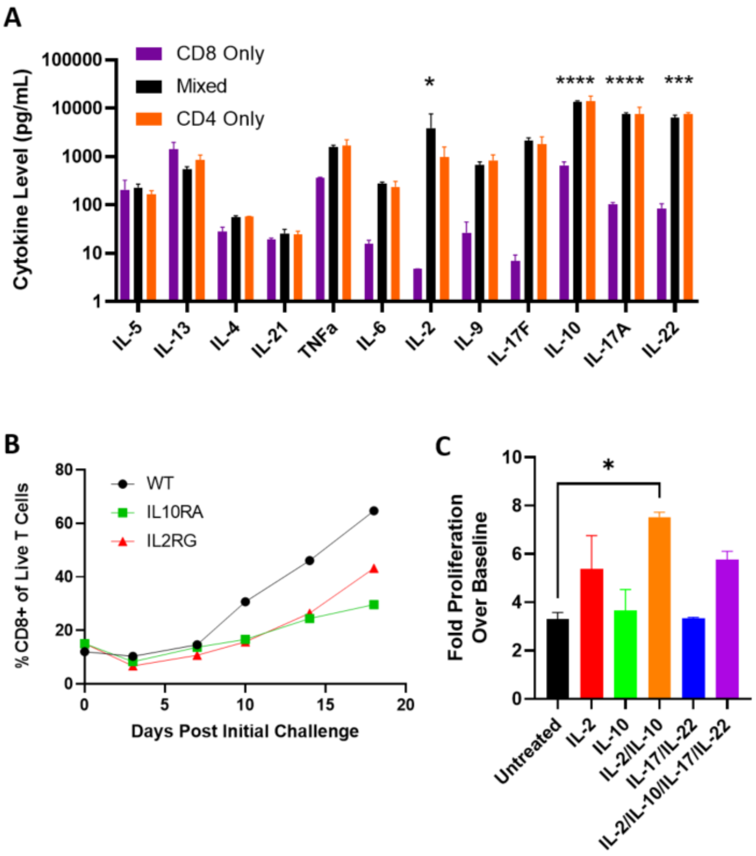

Supplementary Figure 2. CD8+ T cells require CD4+ T cell-derived cytokine help to maintain BsAb-mediated proliferation and central memory-like responses. A. CD4+ and CD8+ T cells were negatively enriched from healthy human donor PBMCs from two separate donors and were either mixed together (4:1; Mixed) or maintained separately. Cells were then challenged with KMS11 target cells (E:T – 1:2) and a CD3-engaging anti-BCMA BsAb for 3 days. Supernatants were then collected and levels of the indicated T cell-derived cytokines therein were quantified using a bead-based 13-plex kit. Data were compared using a two-way ANOVA, with significance being shown for comparisons of CD8+ and mixed T cells (*P<0.05, ***P<0.001, ****P<0.0001). **B.** *IL10RA* or *IL2RG* were knocked out in CD8+ T cells from a healthy human donor and then combined with CD4+ T cells from the same donor at a 4:1 CD4:CD8 ratio. WT CD8+ T cells were similarly cultured as a control. Cells were then cultured in a long-term rechallenge assay, with the frequency of CD8+ T cells being assessed by flow cytometry at each rechallenge time point as indicated. **C.** CD8+ T cells from 2 healthy donors were cultured for 7 days in a repeat challenge assay as in (Fig. S1A), with cell culture supernatants being supplemented with 2.5 ng/mL of the indicated recombinant human cytokines (Peprotech, NJ, USA). Fresh cytokines were added when cells were plated for rechallenge on Day 4, with final proliferation being assessed on day 7. Data were compared via one-way ANOVAs. *P < 0.05. All analyses were independently repeated using T cells from a minimum of 2 donors.

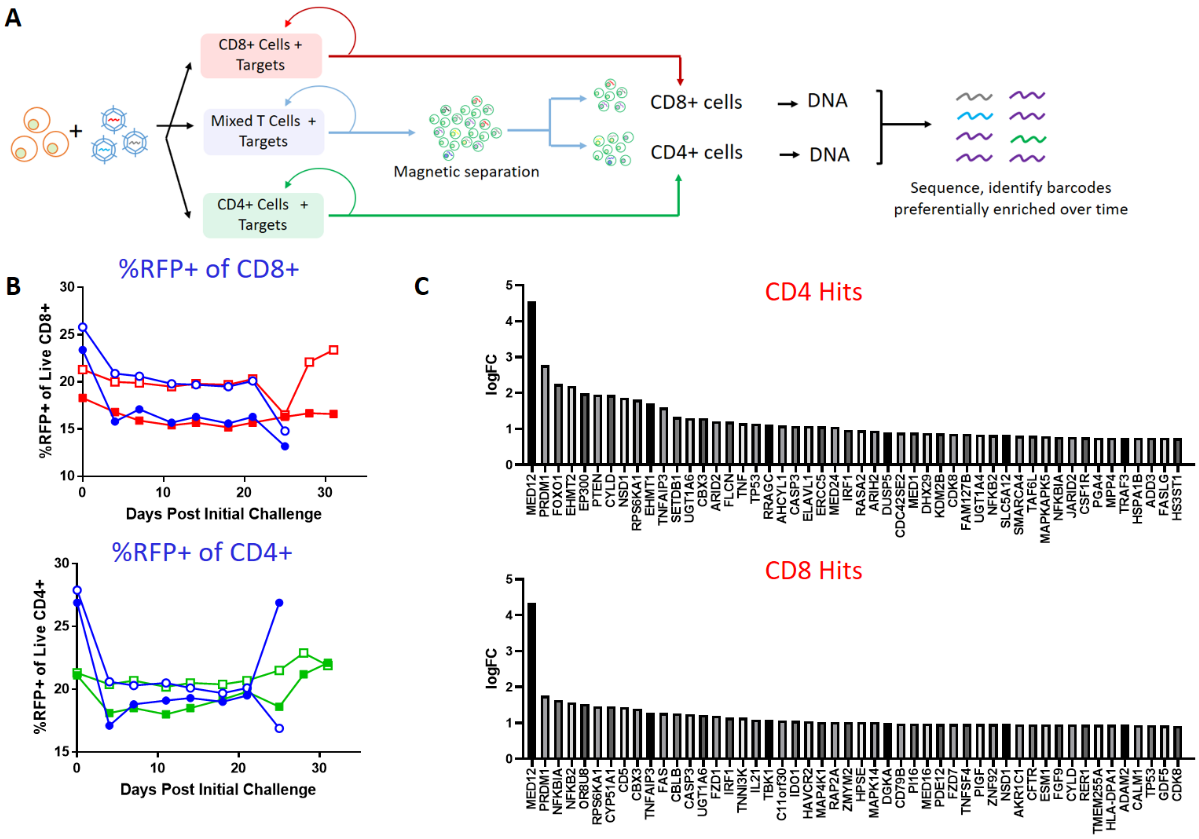

Supplementary Figure 3. CRISPR/Cas9 T cell screening identifies hits specific to CD4+ and CD8+ T cells. A. CRISPR screening was conducted as outlined in Figure 3, with CD4+ or CD8+ T cells from two healthy human donors being screened either in isolation or in combination with one another at a 4:1 CD4:CD8 starting ratio. At collection time points, CD4+ and CD8+ T cells were isolated via positive bead-based magnetic selection, and DNA was then isolated from these cells for sequencing. **B.** The frequencies of RFP+ CD4+ and CD8+ T cells were monitored at each rechallenge time point by flow cytometry. Individual lines correspond to individual donors, and line colors correspond to the color scheme shown in (A). **C.** The top 50 most enriched hits identified in CD4+ and CD8+ T cells were identified based on the average log fold-change (logFC) enrichment values in the CD4+ and CD8+ populations on Days 14 and 21 of culture, with graphed values corresponding to the average logFC values from two donors and two culture conditions (monoculture or co-culture).

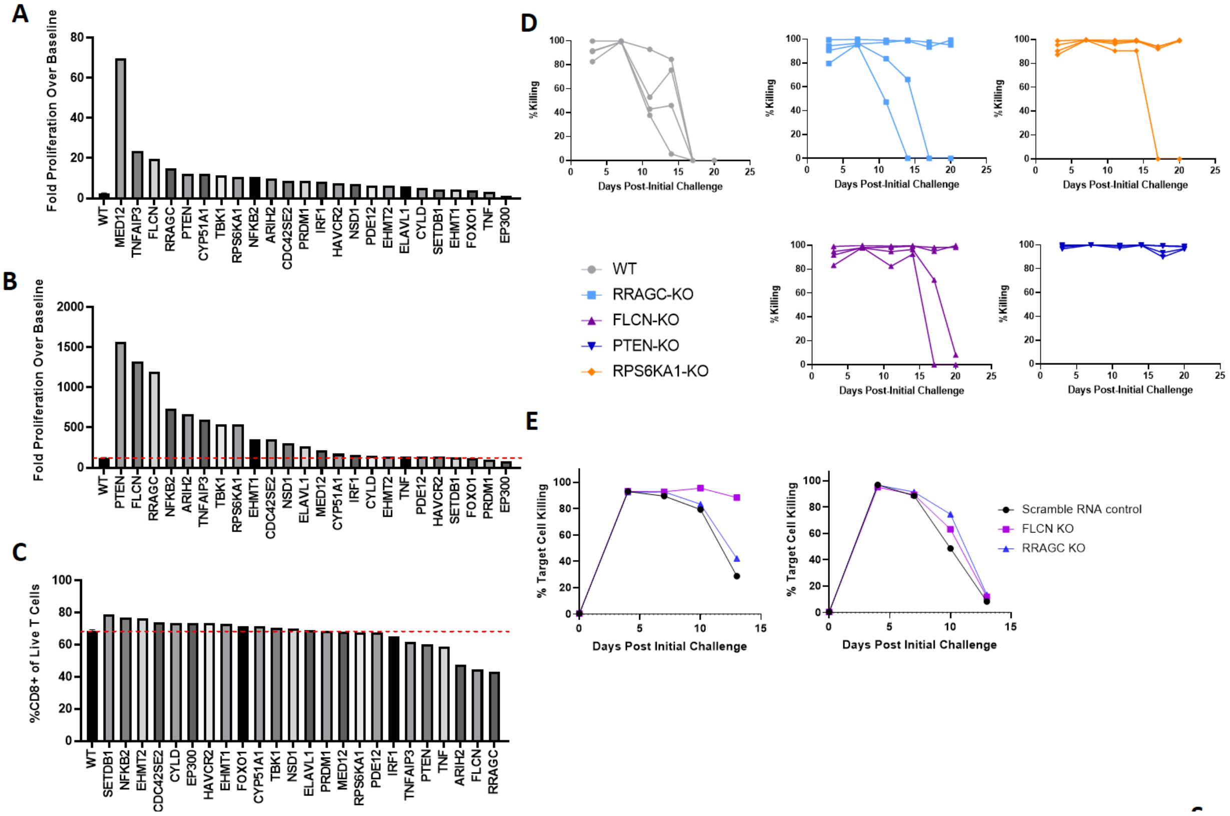

Supplementary Figure 4. Validation of CRISPR screening hits associated with enhanced BsAb-induced T cell proliferation. A-B. A 24-gene validation study was conducted as discussed in Figure 3. The fold proliferation of (**A**) CD8+ T cells at the end of challenge 4 over baseline and (**B**) CD4+ T cells at the end of challenge 5 over baseline was determined via flow cytometry. Gene names correspond to the genes that were individually knocked out in cells via a CRISPR/Cas9 approach. **C.** The frequency of CD8+ T cells among total T cells with the indicated genes having been knocked out at the end of challenge 4 in the repeat challenge validation assay experiment. **D.** KMS11 cell killing by the indicated T cell populations was monitored over time by flow cytometry. Individual plots correspond to data compiled in Fig. 5B. **E.** FLCN or RRAGC were knocked out in total T cells from two healthy human donors. Cells were then used in a repeat challenge assay using adherent HCC1954 target tumor cells. Target cell killing was monitored at each rechallenge time point via flow cytometry after collecting cells from individual wells of a 12-well plate. Individual plots correspond to T cells from two separate donors. Data in A-C were representative of two validation experiments performed in parallel. Data in D are representative of duplicate experiments.

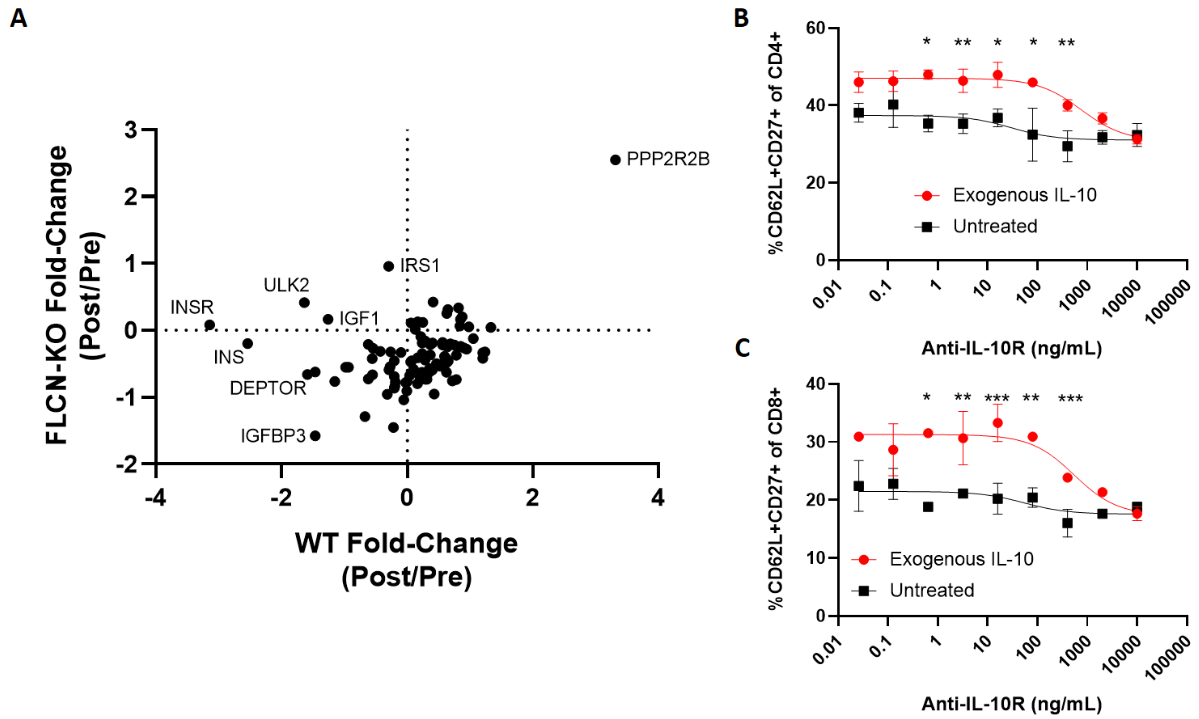

Supplementary Figure 5. Analysis of the roles of FLCN and IL-10 in regulating T cell responses to BsAb-mediated activation. A. RNA samples were collected from WT or *FLCN*-KO T cells from two donors at the end of Challenge 1 or Challenge 4, as shown in Figure 5. Gene expression in these samples was then assessed using an 84-gene mTOR signaling RT^2 Profiler PCR Array (Qiagen) based on provided directions. Data are averages of fold-change in expression values at Challenge 4 relative to Challenge 1 for two donors. **B-C.** T cells from two healthy human donors were combined with KMS11-luc target cells and J6M0 BsAb (3 nM) in the presence of IL-10 (0 or 10 ng/mL) and/or anti-IL-10R (0 – 5 ug/mL in 1:5 dilutions). On day 4 of co-culture, cell surface CD62L and CD27 expression was assessed by flow cytometry and shown for CD4+ T cells (**B**) and CD8+ T cells (**C**) separately. Analyses shown in B and C were repeated in duplicate. Data at each time point in B and C were compared via two-way ANOVAs (*P<0.05, **P<0.01, ***P<0.001).

Gene	crRNA
ARIH2	GCAAGTGAACTGGTACTCCT
EHMT1	AGAAGTCTGCACAAAGTCGT
EHMT2	CGGGCCAAGATGTCAATGAC
ELAVL1	GGAGTGCCACGTTTTTGTTC
EP300	CCTAACCTCAATATGGGAGT
FLCN	GGATCTACCTCATCAACTCC
FOXO1	GGGTCGATCTCCACCACCTG
NSD1	TTGGATTGACCATTACCGAA
PTEN	CTACCTGTTAAAGAATCATC
RPS6KA1	TGAAGAGGTCCCCACCACGC
SETDB1	AAGGAAAGAGTCTACTGTCG
TBCD17	TCGAAGTCCCTCTTTTCCAC
CDC42SE2	GCTGTATGCACAAAGTTTGT
CYLD	TCACTGACGGGGTGTACCAA
CYP51A1	TCAACTACTAGTGCTTGGAT
IL10	GTTGTTAAAGGAGTCCTTGC
IRF1	AAAGTTGGCCTTCCACGTCT
MED12	GGATCTTGAGCTACGAACAC
NFKB2	TAGGCTGTTCCACGATCACC
PRDM1	CATTAAAGCCGTCAATGAAG
RRAGC	CATTCCACAACTGCCGACCT
STAT3	ACAATCCGGGCAATCTCCAT
TNF	TACTCCCAGGTCCTCTTCAA
TNFAIP3	GAATTTACTTGCCTCTCCAC
CBLB	GAGGTCCACCAGATTAGCTC
CD5	AATCATCTGCTACGGACAAC
LCP2	TGAAGAAGTACCACATCGAT
SOCS1	AAGTGCACGCGGATGCTCGT
Nontargetting	CGTTAATCGCGTATAATACG

Supplementary Table 1. crRNAs used for CRISPR validation.

ADDITIONAL FILES

Supplementary Table 2. CRISPR screening sample metadata

Supplementary Table 3. Full CRISPR screening A-N gene level results

Supplementary Table 4. Full CRISPR screening O-Z gene level results
